# Development and application of a novel cervical lymph collection method to assess lymphatic transport in rats

**DOI:** 10.3389/fphar.2023.1111617

**Published:** 2023-01-20

**Authors:** Thu A. Hoang, Enyuan Cao, Gracia Gracia, Joseph A. Nicolazzo, Natalie L. Trevaskis

**Affiliations:** Drug Delivery, Disposition and Dynamics, Monash Institute of Pharmaceutical Sciences, Monash University, Parkville, VIC, Australia

**Keywords:** lymphatics, lymph, cervical, catherization, brain, composition, lipid, protein

## Abstract

**Background:** Fluids, solutes and immune cells have been demonstrated to drain from the brain and surrounding structures to the cervical lymph vessels and nodes in the neck *via* meningeal lymphatics, nasal lymphatics and/or lymphatic vessels associated with cranial nerves. A method to cannulate the efferent cervical lymph duct for continuous cervical lymph fluid collection in rodents has not been described previously and would assist in evaluating the transport of molecules and immune cells from the head and brain *via* the lymphatics, as well as changes in lymphatic transport and lymph composition with different physiological challenges or diseases.

**Aim:** To develop a novel method to cannulate and continuously collect lymph fluid from the cervical lymph duct in rats and to analyze the protein, lipid and immune cell composition of the collected cervical lymph fluid.

**Methods:** Male Sprague-Dawley rats were cannulated at the carotid artery with or without cannulation or ligation at the cervical lymph duct. Samples of blood, whole lymph and isolated lipoprotein fractions of lymph were collected and analyzed for lipid and protein composition using commercial kits. Whole lymph samples were centrifuged and isolated pellets were stained and processed for flow cytometry analysis of CD3^+^, CD4^+^, CD8a^+^, CD45R^+^ (B220) and viable cell populations.

**Results:** Flow rate, phospholipid, triglyceride, cholesterol ester, free cholesterol and protein concentrations in cervical lymph were 0.094 ± 0.014 mL/h, 0.34 ± 0.10, 0.30 ± 0.04, 0.07 ± 0.02, 0.02 ± 0.01 and 16.78 ± 2.06 mg/mL, respectively. Protein was mostly contained within the non-lipoprotein fraction but all lipoprotein types were also present. Flow cytometry analysis of cervical lymph showed that 67.1 ± 7.4% of cells were CD3^+^/CD4^+^ T lymphocytes, 5.8 ± 1.6% of cells were CD3^+^/CD8^+^ T lymphocytes, and 10.8 ± 4.6% of cells were CD3^-^/CD45R^+^ B lymphocytes. The remaining 16.3 ± 4.6% cells were CD3^-^/CD45^-^ and identified as non-lymphocytes.

**Conclusion:** Our novel cervical lymph cannulation method enables quantitative analysis of the lymphatic transport of immune cells and molecules in the cervical lymph of rats for the first time. This valuable tool will enable more detailed quantitative analysis of changes to cervical lymph composition and transport in health and disease, and could be a valuable resource for discovery of biomarkers or therapeutic targets in future studies.

## 1 Introduction

The lymphatic system is a widely distributed network of vessels and lymph nodes that contributes to critical functions in immunity, lipid metabolism, and tissue and fluid homeostasis (Trevaskis et al., 2015b). Structural and functional changes to the lymphatics have been implicated in the pathology of diseases including lymphoedema, cancer metastases, infections, metabolic and immune diseases (Harvey et al., 2005; Proulx et al., 2013; Mortimer and Rockson, 2014; Stacker et al., 2014). In the periphery, entry into the lymphatic vessels from tissues occurs *via* lymphatic capillaries that drain subsequently into pre-collecting lymphatic vessels, larger (afferent) collecting lymphatic vessels, lymph nodes and post-nodal efferent collecting lymphatic vessels. These vessels ultimately join into the right or thoracic lymph ducts from where lymph empties into the blood circulation at the subclavian veins (Oliver et al., 2020; Petrova and Koh, 2020).

In recent years, the presence and function of lymphatics in the central nervous system (CNS) has been the subject of renewed interest following the identification and characterization, using immunohistochemical and imaging techniques, of an extensive network of lymphatic vessels adjacent to vascular sinuses within the dural meninges of mice (Aspelund et al., 2015; Louveau et al., 2015). Subsequently, the meningeal lymphatics have been associated with the pathology of ageing, traumatic brain injury, stroke, brain tumors and neurodegenerative diseases, including Alzheimer’s disease (AD), multiple sclerosis and Parkinson’s disease (Da Mesquita et al., 2018; Louveau et al., 2018; Patel et al., 2019; Zou et al., 2019; Bolte et al., 2020; Hu et al., 2020; Song et al., 2020; Tsai et al., 2022).

The meningeal lymphatic vessels express typical lymphatic endothelial cell (LEC) markers including lymphatic vessel endothelial hyaluronan receptor 1 (LYVE1), vascular endothelial growth factor receptor 3 (VEGFR3), podoplanin (PDPN), chemokine (C-C motif) ligand 21 (CCL21) and prospero homeobox protein 1 (PROX1). They are located along the transverse sinus, sigmoid sinus and other vascular structures in the dura mater, exiting at the base of the brain along blood vessels, along cranial nerves and at the cribriform plate (Aspelund et al., 2015; Louveau et al., 2015). Functional meningeal lymphatic vessels have since been reported in rats, zebrafish, non-human primates and humans (Absinta et al., 2017; Ahn et al., 2019; Castranova et al., 2021).

There has also been extensive effort to characterize other lymphatic outflow routes (in addition to the meningeal lymphatics) from the brain to the cervical lymphatics in the neck. Although it should be noted that robust debate still exists between research groups about the significance of each pathway in transporting fluid and solutes from the brain. Aspelund et al. showed that, following direct injection into the brain parenchyma or cisterna magna, a fluorescent tracer dye (20 kDa IRDye680) drained to meningeal lymphatic vessels at the base of the brain and subsequently to deep cervical lymph nodes (dcLNs) in the neck (Aspelund et al., 2015). Louveau et al. demonstrated that the tracer QDot55 drained to the dcLNs *via* vessels adjacent to superior sagittal sinus following intracisternal injection (Louveau et al., 2015). In contrast, Ma et al. found that different tracers (40 kDa polyethylene glycol [PEG]-ylated IRDye680, Evans blue dye, 3 kDa IRDye680CW or 3 kDa dextran conjugated to Alexa Fluor-680) injected into the lateral ventricles flowed along PROX1^+^ lymphatic vessels near cranial nerves IX, X and XI deriving from the jugular foramen and near cranial nerve VII at the stylomastoid foramen, but not along the dorsal meningeal lymphatic vessels associated with the superior sagittal sinus and transverse sinus. Tracer was also detected at the cribriform plate draining along lymphatic vessels near the olfactory nerve (I), as well as along optic (II) and trigeminal (V) nerves exiting at the base of the skull (Ma et al., 2017). Meningeal lymphatic drainage through the jugular foramen and stylomastoid foramen with connections to extracranial lymphatic vessels has also been confirmed by other researchers with no drainage observed *via* dorsal meningeal lymphatic vessels (Ahn et al., 2019). This study also noted that basal meningeal lymphatic vessels near the petrosquamosal and sigmoid sinuses were characterized by large diameters, extensive capillary branching and lymphatic valves, and thus were more morphologically capable of fluid uptake and transport than the narrower, discontinuous structures of the dorsal meningeal lymphatic vessels found adjacent to the transverse and sagittal sinuses (Ahn et al., 2019).

Despite differing findings regarding the main lymphatic drainage pathway from the CNS between research groups, there is common agreement that the lymphatics predominantly flow from the CNS to the dcLNs (Aspelund et al., 2015; Louveau et al., 2015; Ma et al., 2017; Ahn et al., 2019). On the left side of the head and neck, lymph drains to the left dcLNs and empties into the blood circulation *via* the thoracic lymph duct. On the right side of the head and neck, lymph drains to the right dcLNs and empties, together with lymph from the right arm and upper body, to the blood circulation *via* the right lymph duct (Breslin et al., 2018).

Recent research has indicated that the meningeal and cervical lymphatics perform similar immunological functions to the peripheral lymphatics, including trafficking of antigens and dendritic cells (DCs) to dcLNs leading to T cell activation in mouse models of intracranial tumors, multiple sclerosis and neuroinflammation (Louveau et al., 2018; Hu et al., 2020). In the periphery, lymphatic vessels are also a conduit for lipid and lipoprotein transport. In the intestine, for instance, dietary lipids are packaged into lipoproteins that are transported *via* the mesenteric lymphatics (Bernier-Latmani and Petrova, 2017). High-density lipoproteins (HDLs) also transport cholesterol from peripheral tissues back to the blood circulation *via* the lymphatics (Randolph and Miller, 2014). Interestingly, while smaller HDL-like particles are able to traverse the blood-brain barrier (BBB) into the brain, astrocytes and neurons contribute to the synthesis of the majority of lipoproteins within the CNS (Wang and Eckel, 2014). The trafficking pathways and biological functions of these CNS-derived lipoproteins are under-explored areas of research, and whether meningeal and cervical lymphatics serve as a conduit for lipoprotein transport requires further investigation.

Studies investigating the transport of fluid, fluorescent tracers and immune cells *via* the meningeal and cervical lymphatics in health and disease have predominantly used *in vivo* or *ex vivo* imaging techniques or manipulations such as pharmacological lymphatic ablation and surgical lymph duct ligation (Da Mesquita et al., 2018; Louveau et al., 2018). A method commonly employed to quantify the lymphatic transport of fluid, molecules and immune cells in other tissues is to cannulate the specific lymphatic vessel draining the tissue or organ of interest, such as the mesenteric, hepatic or thoracic lymph ducts into which lymph flows from the intestine, liver and majority of the body, respectively (Bollman et al., 1948; Trevaskis et al., 2015a). These cannulations have mostly been performed in rodents but sometimes in larger species (McHale and Adair, 1989; Kota et al., 2007; Trevaskis et al., 2013).

Herein, we describe a novel method to cannulate the cervical lymph ducts and collect efferent cervical lymph fluid from rats. We used the method to collect and evaluate cervical lymph composition for lipids, proteins and immune cells in rats for the first time. Cervical lymph flow rate and composition was then compared to other lymph sources in the rat. Cannulation of the left and right cervical lymph ducts (also called jugular lymph ducts) has been described in species larger than rats such as rabbits, cats and sheep, though all these studies were performed >20 years ago (Bradbury and Cole, 1980; Boulton et al., 1998). A recent paper from Laura Santambrogio’s group described the collection of afferent cervical lymph (∼1 μL in mice and 2–3 μL in rats) using a pipette or forcep to flush or squeeze out fluid from a section of the lymph duct (Zawieja et al., 2019). However, as far as we are aware, cannulation of the efferent cervical lymph duct to enable continuous collection of the lymph draining the face, head and CNS has not been described previously in small laboratory animals such as rats. This technique has the advantage of enabling collection of larger volumes of lymph as well as enabling quantification of lymph flow and mass transport over time of any molecule of interest in health or disease – be it lipids, vitamins, macromolecules, therapeutics, immune cells or biomarkers. Collection of efferent rather than afferent lymph may also be useful in some contexts (such as, to evaluate different immune cell trafficking pathways). As various lymphatic drainage pathways from the brain converge at the cervical lymphatics, compositional analysis of this lymph fluid can provide precise biochemical and cellular information about the brain’s health. In the future, our novel cervical lymph cannulation technique could therefore be used for a wide range of applications in CNS-related research.

## 2 Materials and methods

### 2.1 Materials

Isoflurane and pentobarbital sodium (Lethabarb^®^) were purchased from Pro-Vet (Victoria, Australia). Heparin sodium was purchased from Hospira (Victoria, Australia). Saline was purchased from Baxter International Inc. (New South Wales, Australia). Polyethylene cannulas (0.96 mm I.D. x 0.58 mm O.D., 0.80 mm O.D. x 0.5 mm I.D. and 0.5 mm O.D. x 0.2 mm I.D.) were purchased from Microtube Extrusions (New South Wales, Australia). All surgical tools, with one exception, were purchased from Fine Science Tools (United States): 2 x straight forceps (11150-10), 2 x haemostats (91308-12), 1 x iris scissors (91500-09), 2 x blunt forceps (11152-10). 1 x fine-tipped titanium forceps (TY-1301) was purchased from ProSciTech (Australia). All fluorescent labelled antibodies and viability dye for flow cytometry were purchased from Thermo Fisher Scientific (Victoria, Australia).

### 2.2 Methods

#### 2.2.1 Animals

Animal studies were approved by the Monash Institute of Pharmaceutical Sciences Animal Ethics Committee and were conducted in accordance with the Australian and New Zealand Council for the Care of Animals in Research and Teaching guidelines. Male Sprague-Dawley rats (7–15 weeks old) were used for the studies and were given free access to water and a standard diet prior to studies.

#### 2.2.2 Pre-surgical preparation

Surgical tools were set out and cleaned overnight by placing in diluted chlorhexidine gluconate 0.1% w/v in water. These tools consisted of 2 x straight forceps, 2 x fine-tipped titanium forceps, 2 x hemostats, 1 x iris scissors and 2 x blunt forceps. A 30 cm section of polyethylene cannula was also pre-soaked in 2.5 I.U./mL heparin in saline at least overnight to prevent clot formation in the cannula. The polyethylene cannula tip was beveled at 45° to aid insertion into the lymph duct.

Prior to surgery, rats were placed under anaesthesia using inhaled isoflurane delivered *via* a nose cone (induction at 5% isoflurane, then maintenance at 1.5%–2.5% isoflurane in medical Carbogen [5% carbon dioxide/95% oxygen]). Rats were laid in a supine position on a heated pad maintained at 37°C. The rats remained anaesthetized for the remainder of the study until they were euthanized with 325 mg sodium pentobarbitone *via* intraperitoneal injection at the conclusion of the study. To prepare the surgical area for cervical lymph cannulation, the neck area on the underside of the rat was shaved and cleared of loose fur.

#### 2.2.3 Cervical lymph and duodenum cannulation

**FIGURE 1:**
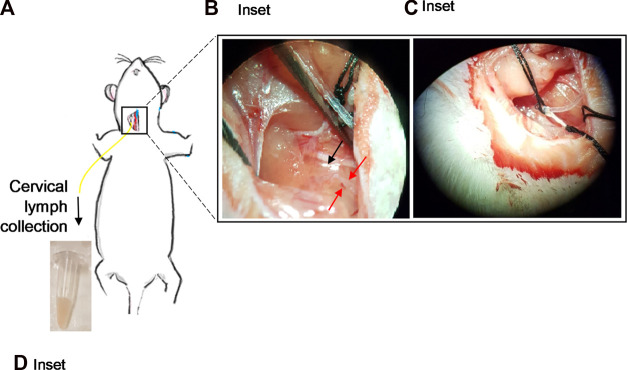
Cannulation of the cervical lymphatic duct in an anaesthetized rat. **(A)** Illustration of an anaesthetized rat showing the surgical site for cervical lymph cannulation. Yellow line represents cannula inserted into the cervical lymph duct. Inset: **(B)** Separation of the muscles around the cervical lymph duct using straight forceps. Red arrows denote location of cervical lymph duct efferent to dcLN. Black arrow denotes the location of the dcLN. **(C)** Insertion of the cannula into the cervical lymph duct. **(D)** Cervical lymph fluid draining from the cannula is collected into pre-weighed vials.

An initial midline incision (∼2 cm in length) was made through the skin from above the larynx to the sternum using a scalpel (Figure 1A). The underlying connective tissue was gently pulled apart using blunt forceps. The muscle layers were then cleared by blunt dissection using blunt forceps, with the sternomastoid muscle gently pushed aside. With the aid of a microscope, the cervical lymph trunk was visualized as a vessel full of faintly white fluid, lying adjacent to the carotid artery. The cervical lymph trunk was also identified as emerging from one or more dcLN(s). If needed, straight forceps and/or hemostat(s) were used to hold back muscle layers and open access to the area (Figure 1B). A pair of iris scissors or fine-tipped forceps was then used to make a small hole across the upper third of the cervical lymph duct. The cannula tip (held by straight or fine-tip forceps) was inserted <0.5 cm into the lymph duct without force, in the direction towards the head (Figure 1C). Once inserted, the cannula was secured with either one or two drop(s) of cyanoacrylate adhesive and/or one or two suture(s) over the site of cannulation. A cannula was also inserted into the duodenum for rehydration with saline as per previously described procedures (Trevaskis et al., 2015a). A continuous infusion of saline (1.5 mL/h) was administered into the duodenum *via* the cannula. Hydration continued throughout the experiment.

#### 2.2.4 Lymph collection

Lymph samples were collected continuously into pre-weighed tubes containing 10 µL of 1000 I.U./mL heparin that were replaced every h for up to 6 h (Figure 1D). The volume of lymph collected was determined gravimetrically to calculate lymph flow rate. Freshly collected lymph was either immediately processed for flow cytometry analysis or stored at -20°C for other subsequent analyses.

#### 2.2.5 Isolation of lipoprotein fractions in lymph samples

In order to separate the cervical lymph into lipoprotein fractions, lymph samples were layered under discontinuous density layers formed by potassium bromide (KBr) salt solutions in an ultracentrifuge tube as described previously (McIntosh et al., 1999; Gracia et al., 2020). After centrifugation, the bottom of the sample tube was punctured with a 25G needle and the separated lipoproteins were allowed to elute into 200 µL fractions, collected into tubes (approximately 20 tubes).

#### 2.2.6 Lipid and protein composition of lymph

The concentrations of triglyceride, phospholipid, free and total cholesterol, and protein in whole cervical lymph and its fractions were determined using the following commercial assay kits: TG GPO-PAP kit from Roche (New South Wales, Australia), Phospholipase C assay kit from Wako Diagnostics (Osaka, Japan), Amplex Red Cholesterol assay kit from Thermo Fisher Scientific, (Victoria, Australia) and bicinchoninic acid (BCA) assay kit from Thermo Fisher Scientific (Victoria, Australia), respectively. The manufacturers’ protocols were followed without modification. Cholesteryl ester concentration was determined by deducting free cholesterol concentration from total cholesterol concentration.

Lymph to plasma protein concentration ratio was calculated using the following equation:

Lymphplasma protein ratio=CLCP

where C_L_ is the total protein concentration in the lymph and C_P_ is the total protein concentration in the plasma (obtained from data previously published by our lab) (Gracia et al., 2020).

#### 2.2.7 Flow cytometry analysis of lymphocytes

Total cells in lymph samples were counted using a LW Scientific hemocytometer (GreyMed, Australia). Additionally, to prepare samples for flow cytometric analysis, a 50 µL volume of lymph was centrifuged at 1800 x g for 3 min and the supernatant removed. The pelleted cells were washed with 200 µL of phosphate buffered saline (PBS, 1 X, pH 7.4) twice and centrifuged at 1800 x g for 3 min between washings. Cells were incubated for 15 min at room temperature with 0.5 µL of 0.2 mg/mL APC mouse anti-rat CD3 (17-0030-80), 0.5 µL of 0.5 mg/mL FITC mouse anti-rat CD4 (11-0040-81), 0.5 µL of 0.2 mg/mL PE mouse anti-rat CD8a (12-0084-80), 0.5 µL of 0.2 mg/mL PE-Cy7 mouse anti-rat CD45R (B220, 25-0460-80) and 0.2 µL of eFluor506 viability dye (65-0866-14) in 200 µL of PBS. Cells were centrifuged, supernatant removed, then washed with 200 µL of PBS once. Cells were then fixed with 200 µL of Becton-Dickinson CytoFix/Cytoperm solution (Thermo Fisher Scientific, United States) for 10 min. Cells were centrifuged, washed and resuspended in 200 µL of PBS, and stored at 4°C until flow cytometric analysis.

Flow cytometric analysis was performed using a Becton-Dickinson FACS Canto II (United States) equipped with 3 laser excitations (488 nm, 633 nm and 405 nm). Unstained negative and single-stained controls were used to set compensation parameters for individual samples. Cells were subsequently identified with characteristic light scatter patterns and then viable cells were identified from negative staining in the V500 channel. CD3^+^/CD4^+^ T lymphocytes from positive staining in the APC and FITC channels, CD3^+^/CD8^+^ T lymphocytes from positive staining in the APC and PE channels, and CD3^-^/CD45R^+^ B lymphocytes from negative staining in the APC channel and positive staining in the PE-Cy7 channel. Fluorescence data was analyzed by FlowJo software (Tree Star, Inc). Flow cytometric plots and detailed gating strategies are provided in Supplementary Figure S1.

#### 2.2.8 Calculations

The concentrations of protein, triglyceride (TG), phospholipid (PL), free cholesterol (FC) and cholesterol ester (CE) in whole lymph were calculated as % mass of each component divided by the total mass of all aforementioned components in whole lymph. Individual lipid concentrations were also calculated as % mass of each lipid component divided by the total mass of all lipid components in whole lymph. The distribution of each component in the various lipoprotein fractions was determined from the % mass of each component in specific lipoprotein fractions divided by mass of each component in all fractions.

#### 2.2.9 Statistical analysis

Statistical analysis was conducted with GraphPad Prism Version 9.2.0 for Windows (GraphPad Software, Inc., CA, United States). Data are presented as mean ± standard error of mean (SEM), unless otherwise specified.

## 3 Results

### 3.1 Optimization of the cervical lymph cannulation method

Cervical lymph duct cannulation was successfully established in a set of anaesthetized rats (n = 5). The cannulation was conducted on the right cervical lymph duct for all animals in this project due to operator preference (e.g., being right-handed and the positioning of animal for surgery). From our experience conducting these studies, a number of factors were critical to ensure the patency of the cannula and extended collection of cervical lymph for up to 8 h. Generally, the larger 0.8 mm O.D. x 0.5 mm I.D. cannula was the preferred size for this procedure as it had less resistance to flow and thus reduced the risk of clotting within the cannula lumen than the narrower 0.5 mm O.D. x 0.2 mm I.D. cannula. However, due to anatomical variability of lymphatic vessel diameters between animals, some ducts were too narrow for the larger cannula. To overcome the limitations associated with a narrow duct, it was occasionally possible to insert a 0.5 mm O.D. x 0.2 mm I.D. cannula tip into the vessel, with a wider 0.8 × 0.5 mm cannula joined to the distal end of the inserted cannula.

Clots and air bubbles were identified *via* visual inspection of the cannula and a reduction in lymph flow rate was sometimes a sign of their presence. To minimize the risk of clotting, cannulas were pre-filled with 2.5 I.U./mL of heparin in saline at least overnight prior to the surgery and were inspected and confirmed to be free of air bubbles prior to insertion. In the event that a clot formed, it was occasionally possible to break up the clot by gently massaging the section of clotted cannula with light pressure between two fingers. Care was taken not to apply too much pressure which could lead to complete occlusion of the cannula.

Furthermore, it was possible for cannulas to become occluded during the application of tissue adhesive. To prevent this from occurring, application of the tissue adhesive after cannula insertion was only conducted while a syringe (containing 2.5 I.U./mL of heparin in saline) was connected to the distal end of the cannula. The syringe was not removed from the cannula end until the tissue adhesive had set.

### 3.2 Flow rate, protein and lipid concentrations of cervical lymph in rats

Following successful cannulation of the cervical lymph vessel, the collected fluid was faintly cloudy and milky (Figure 1D). The mean lymph flow rate through the right cervical lymph duct was 0.094 ± 0.014 mL/h or 0.32 ± 0.08 mL/h/kg (n = 4).

**FIGURE 2:**
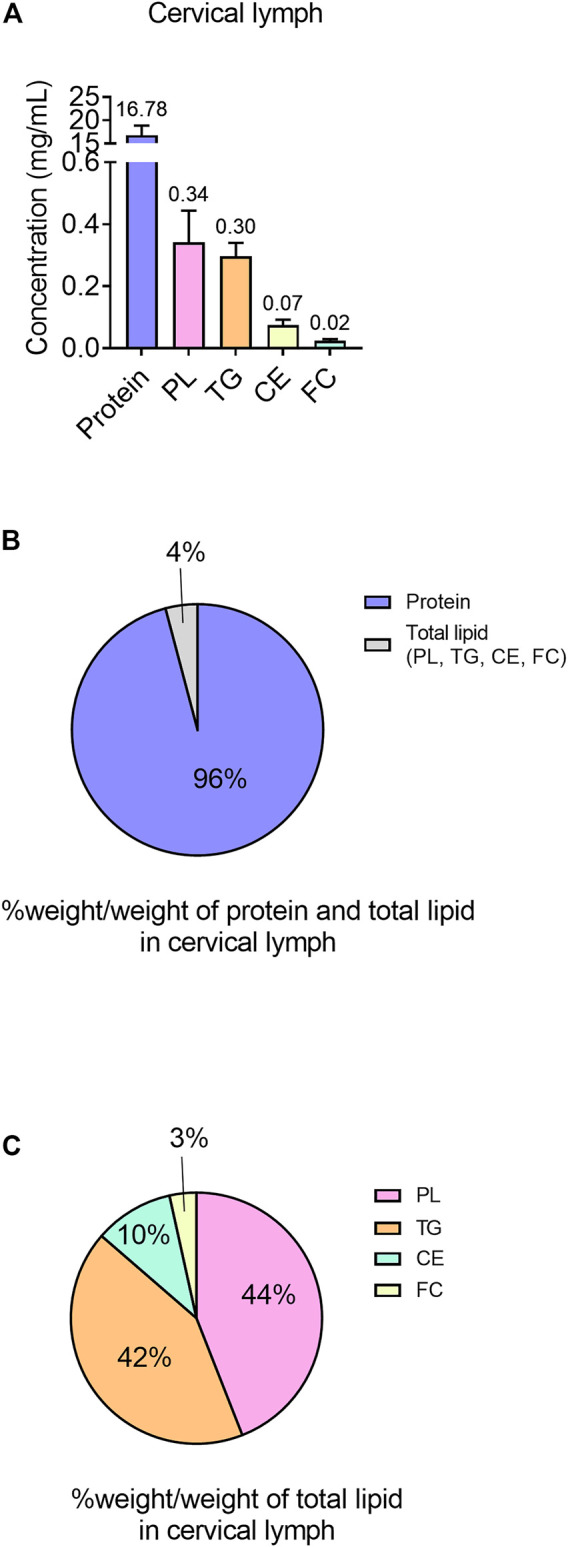
Protein and lipid content in cervical lymph in anaesthetized, non-fasted rats. **(A)** Concentrations (mg/mL) of protein and different lipid types (TG, PL, CE and FC) in lymph. **(B)** Content of protein and lipids (TG, PL, CE and FC) in cervical lymph, expressed as % weight of each component (g)/total weight of protein and total lipids (g). **(C)** Content of lipids (PL, TG, CE and FC) in total lipid component of lymph, expressed as % weight of each component (g)/total weight of total lipids (g). Data presented as mean ± SEM (n = 3). PL = phospholipid; TG = triglyceride; CE = cholesterol ester; FC = free cholesterol.

The mean protein concentration of the cervical lymph was 16.78 ± 2.06 mg/mL (Figure 2A). Furthermore, the mean lymph to plasma protein concentration ratio was 0.21. Cervical lymph had higher concentrations of protein than lipid with 95.8% w/w of total protein and lipid consisting of protein (Figures 2A, B).

The lipid component of cervical lymph mainly consisted of PL (0.34 ± 0.10 mg/mL in whole lymph or 44% w/w of total lipid) and TG (0.30 ± 0.04 mg/mL in whole lymph or 42% w/w of total lipid), with the remaining lipids (<14% w/w) composed of CE (0.075 ± 0.017 mg/mL) and FC (0.024 ± 0.006 mg/mL) (Figures 2A, C).

### 3.3 Distribution of protein and lipid within lipoprotein fractions of cervical lymph

**FIGURE 3:**
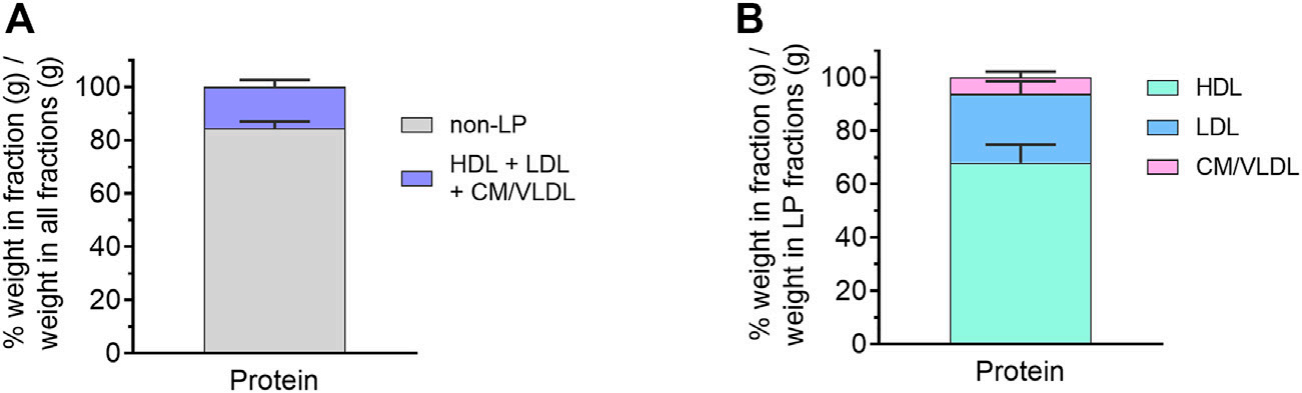
Protein content in isolated lipoprotein (LP) fractions of cervical lymph from anaesthetized, non-fasted rats. **(A)** Distribution of protein in different LP and non-LP fractions. **(B)** Distribution of LP-associated protein in different LP fractions. The protein content in each LP fraction is expressed as % weight in LP (g)/weight in all fractions (g). Data presented as mean ± SEM (n = 3). CM = chylomicron; VLDL = very low-density lipoprotein; LDL = low-density lipoprotein; HDL = high-density lipoprotein.

The distribution of protein and lipid in each lipoprotein (LP) fraction was then evaluated, expressed as % weight (g) in LP/weight (g) in all fractions (Figure 3A). Unfortunately, due to the low volume of lymph collected, individual lipids (TG, PL, CE, FC) were mostly below the limit of quantitation in individual lipoprotein fractions. Nonetheless, protein was predominantly concentrated in the non-LP fraction (84.4 ± 2.5% w/w), suggesting most of the proteins in cervical lymph were soluble proteins (e.g., albumin). The remaining proteins were associated with lipoproteins including HDL (10.4 ± 1.3% w/w of all fractions or 68.0 ± 6.8% w/w of LP fractions only), low-density lipoproteins (LDL, 4.1 ± 1.1% w/w of all fractions or 25.8 ± 4.9% w/w of LP fractions only) and combined TG-rich chylomicrons (CM) and very low-density lipoproteins (VLDL) (1.1 ± 0.5% w/w of all fractions or 6.3 ± 2.2% w/w of LP fractions only) (Figure 3B). These were most likely LP-associated apo-proteins.

### 3.4 Immune cell populations in rat cervical lymph

**FIGURE 4:**
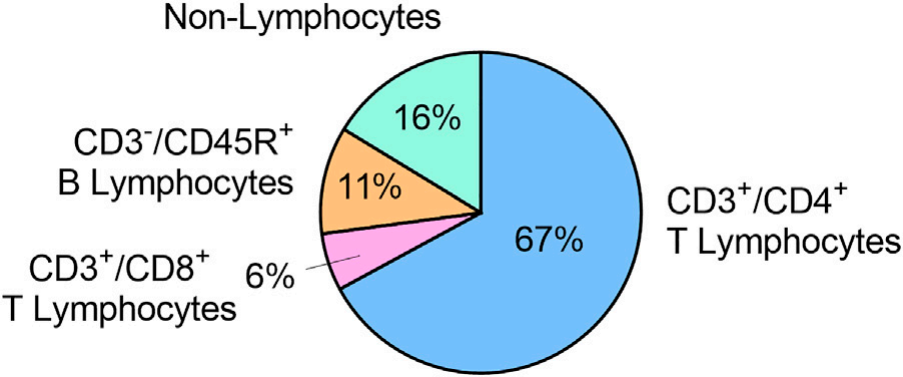
Immune cell populations in cervical lymph fluid, expressed as mean % of total viable cells (n = 5).

The concentration of viable cells in cervical lymph was 7.5 x 10^6^ ± 3.0 x 10^6^ cells/mL (n = 5). In these samples, 67.1 ± 7.4% of cells were CD3^+^/CD4^+^ T lymphocytes, 5.8 ± 1.6% of cells were CD3^+^/CD8^+^ T lymphocytes, and 10.8 ± 4.6% of cells were CD3^-^/CD45R^+^ B lymphocytes. The remaining 16.3 ± 5.8% cells were CD3^-^/CD45^-^ and identified as non-lymphocytes (Figure 4).

## 4 Discussion

### 4.1 Establishing the cervical lymph cannulation method in rats

As far as we are aware, this is the first study to describe the successful cannulation and continuous collection of lymph from the efferent cervical lymph duct in rats. The efferent cervical lymph drains from the dcLNs, which collect lymph from the entire ipsilateral half of the head and neck. Thus, cervical lymph collection *via* cannulation has far-reaching potential to be applied in future rat model-based experiments, wherein lymphatic transport of fluid, cells and any molecule of interest from the brain can be evaluated in baseline and chosen disease states. The collected lymph can be analyzed to study specific biological transport pathways or identify biomarkers and treatment targets for diseases of the brain, head or neck.

Our cervical lymph cannulation method may be preferred over other techniques to study lymphatic transport, such as surgical or pharmacological lymph duct ligation or ablation, and/or single timepoint collection of lymph fluid or node(s), as it allows for continuous *in vivo* collection of lymph. This enables the quantification of lymph transport rate and changes in lymph composition over time in the same animal. Relative to larger animals, the use of rat models for lymph cannulation has the advantage of reduced financial costs, including costs related to animal acquisition, welfare maintenance and associated surgical equipment and resources. Furthermore, the rat cervical lymph cannulation model can be used to investigate changes to lymphatic transport in a wide range of settings and diseases using well-established procedures and disease models in rats.

Some limitations of the cervical lymph cannulation method were, however, noted. It is an invasive procedure and may be difficult to maintain lymph collection over an extended period of time, with success being dependent on the operator’s skill as well as differences in lymph duct anatomy (such as vessel diameter) between animals. In our own study, we had mixed success rates (∼1:3 success to failure ratio), therefore we predict lymph collection failures are likely, if not inevitable, in future studies utilizing this technique. Cannulation of the lymphatic duct also has the potential to alter lymph drainage patterns due to disruption of the normal pressure gradient after cannulation (Kassis et al., 2012). The cervical lymph receives lymph not only from the CNS but also from other lymphatic vessels draining the head and neck, such as those draining the mouth and nasal cavity. This needs to be kept in mind when analyzing the biochemical and cellular composition of the collected cervical lymph.

In our studies, lymph from the right cervical lymph duct was collected. However, it may be possible to collect cervical lymph from both sides in the future to evaluate drainage patterns from different regions of the brain and CSF.

### 4.2 Cervical lymph flow rate is allometrically scaled across rats and other species

The lymph flow rate we measured *via* cannulation of the right cervical lymph duct in rats (0.093 ± 0.014 mL/h) was, as expected, less than that reported previously in larger animals such as sheep (9.1 ± 3.3 mL/h, sum of both left and right cervical lymph ducts), cats (0.3 mL/h, sum of both ducts) and rabbits (0.138 mL/h, one side only) (Bradbury and Cole, 1980; Boulton et al., 1998). In previously conducted studies by our laboratory, mesenteric and thoracic lymph flow rates were shown to scale allometrically across species (mouse, rat, dog and human) with an exponent of ∼0.9, i.e. lymph flow rate increases in direct proportion to increases in body mass to the power of 0.9 (Trevaskis et al., 2020). Using the same calculations for allometric analysis (Trevaskis et al., 2020), the scaling exponent for cervical lymph flow rate from one side was 0.8 across species (rats, rabbits, cats and sheep). This suggests cervical lymph flow rate also increases in proportion to body weight across species and that studies conducted in several species may be able to predict cervical lymph flow rate and transport in larger species, including humans. As expected from the allometric exponent, the body mass-normalized cervical lymph flow rate on one side in rats (0.32 ± 0.08 mL/h/kg) was higher than that described previously in larger species - in sheep, cats and rabbits (∼0.15, ∼0.04 and ∼0.05 mL/h/kg, respectively) (Bradbury and Cole, 1980; Boulton et al., 1998).

Within the rat, cervical lymph flow rate (0.09 ± 0.01 mL/h) was less than reported lymph flow rates from the mesenteric lymph duct (0.4–1.3 mL/h or up to 3.4 mL/h/kg) (Tso et al., 1983; Gracia et al., 2020; Trevaskis et al., 2020), and thoracic lymph duct (0.4–1.4 mL/h) (Trevaskis et al., 2015a; Gracia et al., 2020), but comparable to liver lymph flow rate (0.09–0.22 mL/h) (Tso et al., 1983; Lee, 1984; Shiau et al., 1985; Gracia et al., 2020). Given that the rat brain (∼2 g) is lower in weight than the rat liver (∼10 g) this suggests that a larger volume of lymph is produced per organ mass for the brain, which is remarkable given the high level of metabolic processing of the liver (Piao et al., 2013). It should be noted that the level of hydration, fasting and consciousness state differ across published studies and are also likely to impact lymph flow rates.

### 4.3 Protein and lipid concentrations in cervical lymph compared to other lymph sources

Interestingly, protein concentrations in cervical lymph (17 mg/mL) were similar to concentrations reported in the literature for mesenteric lymph (19 mg/mL) (Gracia et al., 2020), but slightly less than those for hepatic lymph (20–43 mg/mL) (Friedman et al., 1955; Tso et al., 1983; Lee, 1984; Gracia et al., 2020), and thoracic lymph (26–37 mg/mL) (Li et al., 2011; Gracia et al., 2020). The mean lymph to plasma protein ratio was determined to be 0.21, which is less than for hepatic lymph (0.26-0.69), mesenteric lymph (0.26) and thoracic lymph (0.34) (Friedman et al., 1955; Tso et al., 1983; Lee, 1984; Gracia et al., 2020). The lymph to plasma protein ratio is greater in the hepatic and intestinal tissues due to the presence of sinusoidal and fenestrated vascular endothelium, and fenestrated (but not sinusoidal) endothelium, respectively, which facilitates increased extravasation of proteins from the plasma into tissues that subsequently access lymph. Thoracic lymph has a relatively high lymph to plasma protein ratio as it collects protein from hepatic and mesenteric lymph as well as lymph in the entire left and lower right regions of the body (Yadav et al., 2018). As expected, the lymph to plasma protein ratio of cervical lymph was observed to be less than hepatic and thoracic lymph, perhaps because entry of proteins into the brain is tightly restricted by the BBB, leading to reduced drainage of total proteins to the cervical lymph. However, other proteins produced within brain tissue, such as amyloid-β and tau, may drain specifically into the lymphatics leading to high concentrations of these individual proteins (Da Mesquita et al., 2018; Patel et al., 2019). Additionally, the cervical lymph receives proteins from other regions of the head and neck where the capillary endothelium is largely continuous, non-fenestrated and non-sinusoidal and therefore less permeable than the capillaries of the intestine and liver.

The proportion of protein relative to total protein and lipid was 96% w/w in cervical lymph, which was comparable to data previously reported by our laboratory for hepatic lymph and plasma (92% w/w and 95% w/w, respectively) but greater than the protein to lipid ratio in mesenteric and thoracic lymph (both ∼80% w/w), likely because these lymph sources receive dietary lipid (Gracia et al., 2020). Cervical lymph lipids were primarily PL and TG (44% and 42% w/w of total lipids, respectively) followed by CE and FC (10% and 3% w/w, respectively). Hepatic lymph has relatively similar proportions of TG and PL (47% and 31% w/w of total lipids, respectively) followed by FC and CE (16% and 5% w/w). In contrast, mesenteric and thoracic lymph have higher proportions of TG (∼75% w/w), followed by PL, FC and CE (∼18, ∼5 and ∼2% w/w, respectively) (Gracia et al., 2020). Cervical lymph concentrations of TG (0.3 mg/mL) were comparable to liver lymph concentrations (0.2–0.8 mg/mL) (Lee, 1984; Gracia et al., 2020), but lower than non-fasted mesenteric and thoracic lymph (3.4 and 4.8 mg/mL, respectively) (Gracia et al., 2020). As dietary lipids are directly transported from the intestine into the mesenteric lymph, which subsequently drains into thoracic lymph (Shiau et al., 1985), TG concentrations and proportion of total lipid would be expected be higher for mesenteric and thoracic lymph than other lymph sources within the rat. Like hepatic lymph, cervical lymph does not directly receive dietary TG, and this may explain why the observed TG concentrations in hepatic and cervical lymph were similar.

### 4.4 Protein distribution within lipoprotein fractions of cervical lymph is similar to hepatic lymph

Across all isolated LP and LP-deficient fractions of cervical lymph, protein was found to be predominantly distributed in the non-LP fraction (84% w/w of protein in all fractions), which is comparable to reported distributions in thoracic, hepatic and mesenteric lymph (80%–82% w/w), but possibly higher than for lymph-intact plasma (75% w/w) (Gracia et al., 2020). The protein in the non-LP fraction of cervical lymph is possibly derived from the brain and less likely to enter lymph following extravasation from blood due to the impermeability of the BBB, as discussed above.

All lipoprotein types, including CM/VLDL, LDL and HDL were present in cervical lymph. LP-associated protein in cervical lymph was primarily concentrated in HDL (68% w/w of LP fractions), which is similar to reported concentrations for hepatic lymph (71%) but higher than those for mesenteric and thoracic lymph (62% and 54%, respectively) (Gracia et al., 2020). Across LP fractions in cervical lymph, 26% w/w of protein was distributed in LDL, which was also similar to reported concentrations in LDL of hepatic, mesenteric and thoracic lymph (21, 19% and 19% w/w). The remaining LP-associated protein in cervical lymph was distributed in CM/VLDL (6%), which was again similar to hepatic lymph (8%), but less than those reported for thoracic and mesenteric lymph (19% and 27%, respectively). This is expected since CM and VLDL formed within intestinal enterocytes are transported first to mesenteric lymph and then thoracic lymph (Randolph and Miller, 2014). The majority of LPs are excluded from entering the brain by the BBB and the blood-cerebrospinal fluid barrier (Vitali et al., 2014). While plasma LDL and HDL can traverse across the BBB, *via* the LDL receptor (LDLR) and HDL receptor scavenger receptor class B type I (SR-BI) present at the luminal side of the BBB (Dehouck et al., 1994; Rigotti et al., 1997), this route of LP entry into the CNS is contentious (Jin et al., 2019; Van Valkenburgh et al., 2021). Within the brain, astrocytes are considered the primary site for LP production and assembly, with LP components like cholesterol produced by astrocytes and also neurons (Jin et al., 2019). Although their origin has yet to be definitively determined, “HDL-like particles” – which were similar in size and density to plasma HDL – have been identified in CSF (Vitali et al., 2014). It may be possible that some of these LPs drain into the lymphatics surrounding the brain and later to cervical lymph, however, further investigation is required to confirm this.

### 4.5 Immune cell populations of cervical lymph compared to other lymph sources

In the periphery, fluid and immune cells are transported from tissues by afferent lymphatic vessels into LNs, traverse the LNs *via* the subcapsular sinus surrounding the node or *via* lymphatic sinuses and conduits that pass through B and T cell zones that converge at the medullary sinus, before exiting the LN into efferent lymph (Trevaskis et al., 2015b). Immune cells, such as DCs, and a small number of neutrophils, monocytes and macrophages, as well as memory T and B cells can be conveyed by afferent lymphatics to LNs where they can either trap and kill pathogens or present antigens to trigger an adaptive immune response (Hampton and Chtanova, 2019). Alternatively, circulating T and B lymphocytes and neutrophils can extravasate from high endothelial venules (HEVs) directly into LNs (Girard et al., 2012). Immune cells, predominantly naïve lymphocytes, migrate out from the LNs into efferent lymph (Mackay et al., 1990), which drains into the thoracic or right lymph ducts and then into systemic circulation (Trevaskis et al., 2015b).

The proportion of cells in cervical lymph that were CD4^+^ and CD8^+^ T lymphocytes (67% and 6%, respectively) were consistent with the literature values for mesenteric lymph in rats (65%–69% and 10%–12%, respectively) (Trevaskis et al., 2010). However, CD45R^+^ B lymphocytes made up 11% of cells in cervical lymph, which was lower than the reported 22% for “other lymphocytes” (presumably mostly B lymphocytes) in mesenteric lymph (Trevaskis et al., 2010). Conversely, there was a higher proportion of non-lymphocyte cells (16%) in cervical lymph than for mesenteric lymph (<5%) (Trevaskis et al., 2010). Further investigations to identify these non-lymphocytes in cervical lymph may be conducted in the future.

The immune cells in cervical lymph may be partially sourced from the brain *via* meningeal lymphatic drainage. Immunofluorescent staining of mice meningeal lymphatic vessels revealed the presence of CD3e^+^ T lymphocytes, as well as CD11c^+^ cells (likely to be mostly DCs) and B220^+^ cells co-localizing with LYVE1^+^ lymphatic endothelial cells (Louveau et al., 2015). In addition, CD4^+^ T lymphocytes and DCs have been demonstrated to be transported by meningeal lymphatics to dcLNs mediated by C-C chemokine receptor type 7 (Louveau et al., 2018). The immune cells in the cervical lymph may thus have trafficked from the meninges *via* the lymphatics.

## 5 Conclusion

We have developed a novel cervical lymph duct cannulation method in anaesthetized rats, enabling continuous direct collection and quantitative analysis of cervical lymph composition and transport rates. This method has the potential to be a useful tool for a myriad of applications including evaluating the lymphatic transport of a wide range of molecules, lipids/lipoproteins and xenobiotics from the brain. The data generated with respect to the cervical lymph composition provides valuable reference physiological values for cervical lymph components, which can be later used for comparisons to evaluate disease- and age-related changes to lymphatic drainage from the brain.

## Data Availability

The original contributions presented in the study are included in the article/Supplementary Material, further inquiries can be directed to the corresponding author. GraphPad Prism (RRID:SCR_002798) FlowJo (RRID:SCR_008520) BD Scientific Canto II Flow Cytometer (RRID:SCR_018056) (Thermo Fisher Scientific Cat# 17-0030-80, RRID:AB_11217880) (Thermo Fisher Scientific Cat# 11-0040-81, RRID:AB_953579) (Thermo Fisher Scientific Cat# 12-0084-80, RRID:AB_1210760) (Thermo Fisher Scientific Cat# 25-0460-80, RRID:AB_2573351).
