# Supplementary material for: Development and application of a novel cervical lymph collection method to assess lymphatic transport in rats
Source: Front Pharmacol. 2023 Jan 20;14:1111617. doi: 10.3389/fphar.2023.1111617 (PMC9895367; doi:10.3389/fphar.2023.1111617)
Supplement: Supplementary file 1 [file DataSheet1.docx]

Supplementary Material

**Supplementary Figure 1.** Representative flow cytometric plots of cervical lymph with gating strategies for T and B lymphocytes. (A) Gating strategy for CD3^+^/CD4^+^ and CD3^+^/CD8^+^ T lymphocytes. (B) Gating strategy for CD3^-^/CD45R^+^ B lymphocytes.
